# Dietary Fish Oil Inhibits Pro-Inflammatory and ER Stress Signalling Pathways in the Liver of Sows during Lactation

**DOI:** 10.1371/journal.pone.0137684

**Published:** 2015-09-09

**Authors:** Denise K. Gessner, Birthe Gröne, Aline Couturier, Susann Rosenbaum, Sonja Hillen, Sabrina Becker, Georg Erhardt, Gerald Reiner, Robert Ringseis, Klaus Eder

**Affiliations:** 1 Institute of Animal Nutrition and Nutrition Physiology, Justus-Liebig-Universität Giessen, Heinrich-Buff-Ring 26–32, 35392, Giessen, Germany; 2 Department of Veterinary Clinical Sciences, Swine Diseases, Justus-Liebig-Universität Giessen, Frankfurter Strasse 112, 35392, Giessen, Germany; 3 Institute for Animal Breeding and Genetics, Justus-Liebig-Universität Giessen, Ludwigstrasse 21b, 35390, Giessen, Germany; Ohio State University Medical Center, UNITED STATES

## Abstract

Lactating sows have been shown to develop typical signs of an inflammatory condition in the liver during the transition from pregnancy to lactation. Hepatic inflammation is considered critical due to the induction of an acute phase response and the activation of stress signaling pathways like the endoplasmic reticulum (ER) stress-induced unfolded protein response (UPR), both of which impair animal´s health and performance. Whether ER stress-induced UPR is also activated in the liver of lactating sows and whether dietary fish oil as a source of anti-inflammatory effects n-3 PUFA is able to attenuate hepatic inflammation and ER stress-induced UPR in the liver of sows is currently unknown. Based on this, two experiments with lactating sows were performed. The first experiment revealed that ER stress-induced UPR occurs also in the liver of sows during lactation. This was evident from the up-regulation of a set of genes regulated by the UPR and numerically increased phosphorylation of the ER stress-transducer PERK and PERK-mediated phosphorylation of eIF2α and IκB. The second experiment showed that fish oil inhibits ER stress-induced UPR in the liver of lactating sows. This was demonstrated by decreased mRNA levels of a number of UPR-regulated genes and reduced phosphorylation of PERK and PERK-mediated phosphorylation of eIF2α and IκB in the liver of the fish oil group. The mRNA levels of various nuclear factor-κB-regulated genes encoding inflammatory mediators and acute phase proteins in the liver of lactating sows were also reduced in the fish oil group. In line with this, the plasma levels of acute phase proteins were reduced in the fish oil group, although differences to the control group were not significant. In conclusion, ER stress-induced UPR is present in the liver of lactating sows and fish oil is able to inhibit inflammatory signaling pathways and ER stress-induced UPR in the liver.

## Introduction

Lactation is a physiological state, which is characterized by a marked increase in energy and nutrient requirement for production of milk. In most mammals, this elevated energy and nutrient demand is met by an increase in food intake and a mobilisation of body´s energy stores, i.e., white adipose and muscle tissue [1–3]. To conserve energy and metabolic substrates for milk synthesis in the lactating mammary gland, most species develop a diversity of metabolic adaptations in the liver, such as a reduced oxidation of fatty acids through down-regulation of transcriptional regulators of genes involved in fatty acid utilization and export of triacylglycerols from the liver to the lactating mammary gland [4–9]. Besides metabolic adaptations, pathophysiologic conditions are commonly developing in the liver during the transition from pregnancy to lactation. For instance, in dairy cows a pro-inflammatory condition in the liver is arising in early lactation, which has been suggested to be associated with the development of fatty liver syndrome and ketosis [10,11]. While metabolic adaptations and pathophysiologic conditions developing during early lactation have been well studied in dairy cows, little is known about specific, corresponding adaptation processes in sows (*Sus scrofa*). Recently, we have observed that lactation induces also a pro-inflammatory condition in the liver of sows, as evidenced from activation of the key regulator of inflammation nuclear factor-kappa B (NF-κB) and up-regulation of genes encoding positive acute phase proteins (APPs), like haptoglobin (HP) and C-reactive protein (CRP) [12,13]. In line with this, earlier observations showed that the plasma levels of APPs, like HP and CRP, are elevated in sows one week after farrowing compared to late pregnancy [14]. The hepatic production of APPs, which is mediated by pro-inflammatory cytokines and occurs in response to different stimuli including infections, tissue damage and stress [15], is regarded as detrimental in farm animals as it not only increases energy and amino acid requirement in the liver for the synthesis of positive APPs but also commonly impairs liver function [16]. Moreover, the pro-inflammatory cytokines such as tumour necrosis factor (TNF)-α generated during an inflammatory process are able to induce stress of the endoplasmic reticulum (ER), a state in which unfolded or misfolded proteins accumulate in the ER lumen [17,18]. ER stress leads to the activation of an adaptive response known as the unfolded protein response (UPR), which aims to restore ER homeostasis and functions by triggering three kinds of protective cellular responses: (i) up-regulation of ER chaperones to assist in the refolding of proteins; (ii) attenuation of protein translation, and (iii) degradation of misfolded proteins by the proteasome by a process called ER-associated degradation (ERAD) [19,20]. Moreover, an induction of the UPR leads to an enhancement of inflammation by activation of NF-κB, a stimulation of lipid biosynthesis and an induction of fibroblast growth factor (FGF) 21, which is a hormonal regulator of lipolysis and ketogenesis [21–23]. Besides these adverse effects, the UPR leads to an improvement of the antioxidant and cytoprotective capacity by activation of nuclear factor E2-related factor 2 (Nrf2) [24]. In the case that ER stress-induced damage is too strong and homeostasis cannot be restored, the UPR can lead to cell death by the induction of apoptosis [25,26]. Recently, it has been observed that ER stress occurs in the liver of dairy cows during early lactation and it has been suggested that the concomitant UPR might be involved in the development of fatty liver syndrome and ketosis [27]. Whether the inflammatory process in the liver observed in lactating sows also leads to ER stress and induction of UPR in the liver during lactation, however, is currently unknown and remains to be demonstrated.

Both, in humans and experimental animal models, it has been well established that n-3 polyunsaturated fatty acids (PUFA) exert anti-inflammatory properties due to inhibition of the pro-inflammatory transcription factor (NF-κB), induction of an “anti-inflammatory” eicosanoid profile (production of 3- and 5-series eicosanoids at the expense of 2- and 4-series eicosanoids), and the production of anti-inflammatory resolvins [28,29]. In sows, it has been shown that feeding fish oil to sows as a source of n-3 PUFA improves postnatal growth of piglets and reduces pre-weaning piglet mortality [30,31]. Moreover, it has been found that supplementation of sows with n-3 PUFA during lactation leads to an increased litter size in the subsequent parity [32]. However, only few studies have been published so far dealing with potential anti-inflammatory effects of n-3 PUFA in sows. In one of those studies, Papadopoulos et al. [33] were able to show that a diet with a low n-6:n-3 PUFA ratio slightly reduces plasma levels of the APP serum amyloid A (SAA) in sows indicating that n-3 PUFA probably act anti-inflammatory in the liver of sows. To our knowledge, direct evidence that dietary n-3 PUFA inhibit inflammation in the liver of lactating sows has not been provided yet.

Based on this, the present study aimed to test two hypotheses: First, the pro-inflammatory process in the liver of lactating sows leads to ER stress and induction of the UPR. Second, dietary n-3 PUFA exert anti-inflammatory effects in the liver of sows and thus counteract the lactation-induced pro-inflammatory condition and ER stress-induced UPR. In order to investigate whether the occurrence of ER stress during lactation and the potential inhibitory effect of fish oil are tissue-specific, we also considered the skeletal muscle in this study and investigated the effect on ER stress-induced UPR, NF-κB and Nrf2 signaling. We also studied the effect on mRNA levels of genes involved in the NOD-like receptor P3 (NLRP3) inflammasome pathway, a critical pro-inflammatory signaling pathway [34] which has been scarcely investigated in sows so far.

## Materials and Methods

For this study, two trials with sows were performed in accordance with established guidelines for the care and handling of laboratory animals and were approved by the local Animal Welfare Authorities (Regierungspräsidium Giessen; permission no: GI 19/3-No. 29/2010).

### Animals

In experiment 1, which has been described recently in more detail [13], twenty second parity sows (Large White & German Landrace) were used. In brief, the sows were artificially inseminated and fed a commercial diet for gestating sows *ad libitum* throughout pregnancy. At the day of farrowing, the sows were randomly assigned into two groups of 10 animals each. In the first group of sows, all piglets were removed from the sow (“non-lactating group”) 24 h after parturition. This group served as the non-lactating control. In the second group, litters were adjusted to 12 piglets per sow (“lactating group”). Throughout lactation until the end of the experiment the sows received a diet for lactating sows. A full description of the housing condition, diet composition, and feeding regime can be found in our recent publication [13]. In addition, data on daily feed intake, body weight development and energy balance of the sows have been reported there [13].

In experiment 2, twenty second parity sows (Large White and German Landrace) were used and artificially inseminated as described recently in more detail [35]. In brief, after farrowing the sows were randomly divided into two groups (control group and fish oil group) of 10 animals each, and litter sizes were adjusted to 8 piglets per sow and the sows of the two groups received two different diets throughout lactation. In the control group, the diet contained 50 g of a mixture of palm oil and soybean oil (4:1, w/w, both oils were obtained from Henry Lamotte Oils GmbH, Bremen, Germany) per kg, whereas in the fish oil group the diet contained 50 g of fish oil (‘Marine oil’, obtained from Henry Lamotte Oils GmbH) per kg. A full description of the housing condition, diet composition, and feeding regime can be found in our recent publication [35]. In addition, data on daily feed intake, body weight development and energy balance of the sows have been reported there [35]. All efforts were made to minimize suffering. The sows showed no clinical signs of diseases during the trial.

After finishing the experiments all sows were returned into the sow herd of the animal keeping facility.

### Sample collection

In both experiments, at day 20 of lactation, blood from *Vena jugularis* was collected 3 h after feed intake into heparinized polyethylene tubes (Sarstedt, Nürnberg, Germany), and plasma was obtained by centrifugation of the blood (1100 × g, 10 min, 4°C) and stored at -20°C pending analysis. At the same day, liver and skeletal muscle biopsy samples were taken percutaneously after anaesthesia by intravenous injection with 2 mg azaperon (Stresnil, Janssen-Cilag GmbH, Neuss, Germany) per kg body mass, 20 mg ketamine (Ursotamin, Serumwerke Bernburg AG, Germany) per kg body mass and up to 2.4 mg thiopental (Thiopental Inresa 0.5 g, Freiburg, Germany) per kg body mass as required for maintenance of anaesthesia. The biopsy procedure has been described recently in detail [36]. Liver samples were immediately snap-frozen and stored at -80°C pending analysis.

### RNA isolation and qPCR

RNA isolation from frozen liver and muscle samples and quantitative real-time PCR (qPCR) analysis were performed as described in Gessner et al. [37]. Briefly, total RNA from frozen liver and skeletal muscle samples was isolated using Trizol Reagent (Invitrogen, Karlsruhe, Germany) and purified using the RNeasy Minikit (Qiagen, Hilden, Germany). The mRNA was reverse-transcribed using 1.2 μg of total RNA, 100 pmol oligo(dT)18 primer (Eurofins MWG Operon, Ebersberg, Germany), 1.25 μl 10 mM dNTP mix (GeneCraft, Lüdinghausen, Germany), 5 μl 59 RT reaction buffer (Thermo Fisher Scientific, St. Leon-Rot, Deutschland), and 60 units M-MuLVReverse Transcriptase (Thermo Fisher Scientific, Schwerte, Germany) at 42°C for 60 min, and a final inactivating step at 70°C for 10 min in a thermal cycler (Biometra, Gttingen, Germany). The qPCR analysis was performed with a Rotorgene 2000 system (Corbett Research, Mortlake, Australia). Gene-specific primer pairs, which were designed by using PRIMER3 and BLAST, were obtained from Eurofins MWG Operon (Ebersberg, Germany). Primer characteristics of reference genes of both trials were published recently [12,35]. Characteristics of gene-specific primers used for qPCR analysis of target genes are shown in Table 1. Ct-values of target and reference genes were obtained using Rotorgene software 5.0 (Corbett Research) and relative mRNA expression levels were calculated using GeNorm normalisation factor, including the three most stable out of six reference genes [38].

**Table 1:** Characteristics of gene-specific primers used for qPCR.

Gene^1^	Forward primer (from 5`to 3`)	Product length	Accession number
	Reverse primer (from 5`to 3`)		
*NF-κB target genes*			
*CCL2*	CTGCACCCAGGTCCTTGC	199	NM_214214.1
	GACCCACTTCTGCTTGGGTTC		
*HP*	GTTCGCTATCACTGCCAAAC	108	NM_214000.2
	CAGTTTCTCTCCAGTGACCT		
*ICAM1*	CGGTGGCAGCCGTGGCTATC	208	NM_213816.1
	TTGATGCAGCCCCGCTCGTC		
*IL8*	ACTTCCAAACTGGCTGTTGC	120	NM_213867.1
	GGAATGCGTATTTATGCACTGG		
*LBP*	ACCGCTCCCCAGTTGGCTTC	406	NM_001128435.1
	AGCGCGGCGGACACATTAGT		
*PTGS2*	CACCGCAACGCCTCTACC	105	NM_214321.1
	GCAGTGCAGAGCGACACG		
*SAA2*	GGCATCATTCCTCAAGGAAG	168	NM_001044552.1
	CTGATCACTTTAGCAGCCCA		
*TNF*	CATGAGCACTGAGAGCATGA	180	NM_214022.1
	CGATAACCTCGAAGTGCAGT		
*UPR target genes*			
*ATF4*	AACATGGCCGAGATGAGCTTCC	265	NM_001123078.1
	TCTCCACCATCCAGTCTGTCCC		
*BAK1*	AGGACCTGAGAGATGGCGTCC	283	XM_001928147.2
	AGTCGTATCGCCGGTTGATGTC		
*BAX*	ATGGAGCTGCAGAGGATGATCG	289	XM_003127290.3
	ACGTGGGCGTCCCAAAGTAG		
*BCL2L1*	CGTCCCAGCTCCACATCACC	147	NM_214285.1
	CCTTGTCTACGCTCTCCACGC		
*CASP3*	CTGCCGAGGCACAGAATTG	135	NM_214131.1
	CGCCAGGAATAGTAACCAGGTG		
*CASP8*	AGAAAGATGTCCCAGGGGTGAAGA	121	NM_001031779.2
	CAGGGTGAAAGTAGGTTGTGGCA		
*DDIT3*	CTGAGTCATTGCCTTTCTCCTTCG	311	NM_001144845.1
	ACTTTGTTTCCGTTTCCTGGGTC		
*DNAJC3*	TGTCTCTCAGTGAAGTTCGTGAATG	160	NM_001190184.1
	GATTCATATTTGCTGGTCGCATC		
*EDEM1*	TGGGTTGGAAAGCAGAGTGGC	200	XM_005669741.1
	TTCACATTGACGTAGAGTGGCGG		
*HSP90B1*	GCTTGTCCGTAAAACTCTGG	196	NM_214103.1
	CACATACTGGTCTAGACTAGT		
*HSPA5*	TGGAATGACCCGTCTGTGC	120	XM_001927795.5
	TGGTGCAAATGTCTTTGTTTGC		
*PDIA4*	CAATGACGCCAAGCGCTAC	178	NM_001267834.1
	CACCTCCGTGGCGAAGTC		
*PPP1R15A*	GGCAGTAACCAGGGCAGACG	236	XM_003127275.2
	TTCCGGGCTCTCTAGGGACG		
*TP53*	ACTAAGCGAGCACTGCCCAC	155	NM_213824.3
	GTCTGGGCATCCTTCAGCTCC		
*Nrf2 target genes*			
*CYP1A1*	CTGCCATCTTCTGCCTTGTA	314	NM_214412.1
	GCTCTGGCCATTAGAGATCA		
*GPX1*	CTTCGAGAAGTTCCTGGTGG	232	NM_214201.1
	CCTGGACATCAGGTGTTCCT		
*HMOX1*	AGCTGTTTCTGAGCCTCCAA	130	NM_001004027.1
	CAAGACGGAAACACGAGACA		
*NQO1*	CCAGCAGCCCGGCCAATCTG	160	NM_001159613.1
	AGGTCCGACACGGCGACCTC		
*PRDX6*	GGCCGCATCCGTTTCCACGA	280	NM_214408.1
	ACTGGATGGCAAGGTCCCGACT		
*SOD1*	TCCATGTCCATCAGTTTGGA	250	NM_001190422.1
	CTGCCCAAGTCATCTGGTTT		
*TXNRD1*	CTTTACCTTATTGCCCGGGT	162	NM_214154.3
	GTTCACCGATTTTGTTGGCC		
*NLRP3 Inflammasome pathway*			
*CASP1*	GCGTCTTCAGAGCCAAGAGG	137	NM_214162.1
	TTGCAGATTATGAGGGCAAGG		
*IL1B*	GTTCTCTGAGAAATGGGAGC	143	NM_214055.1
	CTGGTCATCATCACAGAAGG		
*NLRP3*	GTTGCACCCGAACTGCAAGC	123	NM_001256770.1
	CCTAGGCTCAGCTTTCGCAGG		
*PYCARD*	GACATCGGCATGAAGGAGGTGG	118	XM_003124468.3
	GCAGTGCTGGTTTGTTGTCTGC		

^1^Abbreviations: *ATF4*, activating transcription factor 4; *BAK1*, BCL2-antagonist/killer 1; *BAX*, BCL2-associated X protein; *BCL2L1*, BCL2-like 1; *CASP*, caspase, apoptosis-related cysteine peptidase; *CCL2*, chemokine (C-C motif) ligand 2; *CYP1A1*, cytochrome P450, family 1, subfamily A, polypeptide 1; *DDIT3*, DNA-damage-inducible transcript 3; *DNAJC3*, DnaJ (Hsp40) homolog, subfamily C, member 3; *EDEM1*, ER degradation enhancer, mannosidase alpha-like 1; *GPX1*, glutathione peroxidase 1; *HMOX1*, heme oxygenase 1; *HP*, haptoglobin; *HSP90B1*, heat shock protein 90kDa beta (Grp94), member 1; *HSPA5*, heat shock 70kDa protein 5 (glucose-regulated protein, 78kDa); *ICAM1*, intercellular adhesion molecule 1; *IL1B*, interleukin 1, beta; *LBP*, lipopolysaccharide binding protein; *NLRP3*, NLR family, pyrin domain containing 3; *NQO1*, NAD(P)H dehydrogenase, quinone 1; *PPP1R15A*, protein phosphatase 1, regulatory subunit 15A; *PTGS2*, prostaglandin-endoperoxide synthase 2; *PRDX6*, peroxiredoxin 6; *PYCARD*, PYD and CARD domain containing; *SAA2*, serum amyloid A2; *SOD1*, superoxide dismutase 1, soluble; *TNF*, tumor necrosis factor; *TP53*, tumor protein p53; *TXNRD1*, thioredoxin reductase 1.

### Western blotting

Homogenates from liver tissue were prepared and protein concentrations determined as described recently [39]. Following protein separation by 12.5% SDS-PAGE the proteins were transferred to a nitrocellulose membrane and incubated with primary antibodies against phosphorylated protein kinase-like endoplasmic reticulum kinase (PERK) (monoclonal anti-phospho PERK antibody; Cell Signaling Technology, Boston, MA, USA), total PERK (polyclonal anti-PERK antibody; Santa Cruz Biotechnology, Inc., Santa Cruz, Ca, USA), phosphorylated α subunit of eukaryotic translation initiation factor 2 (eIF2α) (polyclonal anti-phospho-eIF2α-Ser^51^ antibody, Cell Signaling Technology, Boston, MA, USA), total eIF2α (polyclonal anti-eIF2α antibody, Cell Signaling Technology, Boston, MA, USA), phosphorylated nuclear factor of κ light polypeptide gene enhancer in B-cells inhibitor α (IκBα) (monoclonal anti-IκBα phospho S^32^+S^36^ antibody, Abcam, Cambridge, UK), total IκBα (monoclonal, anti-IκBα antibody, Abcam, Cambridge, UK) and α-tubulin (monoclonal anti-α-tubulin antibody, Cell Signaling Technology, Boston, MA, USA) as a reference protein. The membranes were washed, and then incubated with a horseradish peroxidase conjugated secondary monoclonal anti-mouse-IgG antibody (Sigma-Aldrich, Steinheim, Germany) for phospho-IκBα, total IκBα and polyclonal anti-rabbit-IgG antibody (DakoCytomation, Glostrup, Denmark) for phospho-PERK, phospho-eIF2α, total eIF2α and α-tubulin and polyclonal anti-goat-IgG antibody (Santa Cruz Biotechnology, Inc., Santa Cruz, Ca, USA) for total PERK at room temperature. Afterwards blots were developed by ECL Select (GE Healthcare, Munich, Germany) and the intensities of the specific bands were detected with a Bio-Imaging system (Syngene, Cambridge, UK) and quantified by Syngene GeneTools software (nonlinear dynamics).

### Determination of plasma concentrations of HP und CRP

Plasma concentrations of HP and CRP were determined by Phase Range kits from Tridelta Development Ltd. (Maynooth, Co. Kildare, Ireland; cat. no. TP801 and TA901 for HP kit and CRP kit, respectively).

### Statistical analysis

All data were tested for normal distribution by Shapiro-Wilk following testing for detection of outliers. Means of the two groups in each of the experiments were compared using student’s t-test for parametric variables, and Kruskal-Wallis test for nonparametric variables. Differences between means were considered statistically significant for p<0.05.

## Results

### Relative mRNA concentrations of genes involved in the UPR in the liver and skeletal muscle of lactating and non-lactating sows on day 20 of lactation

**Fig 1:**
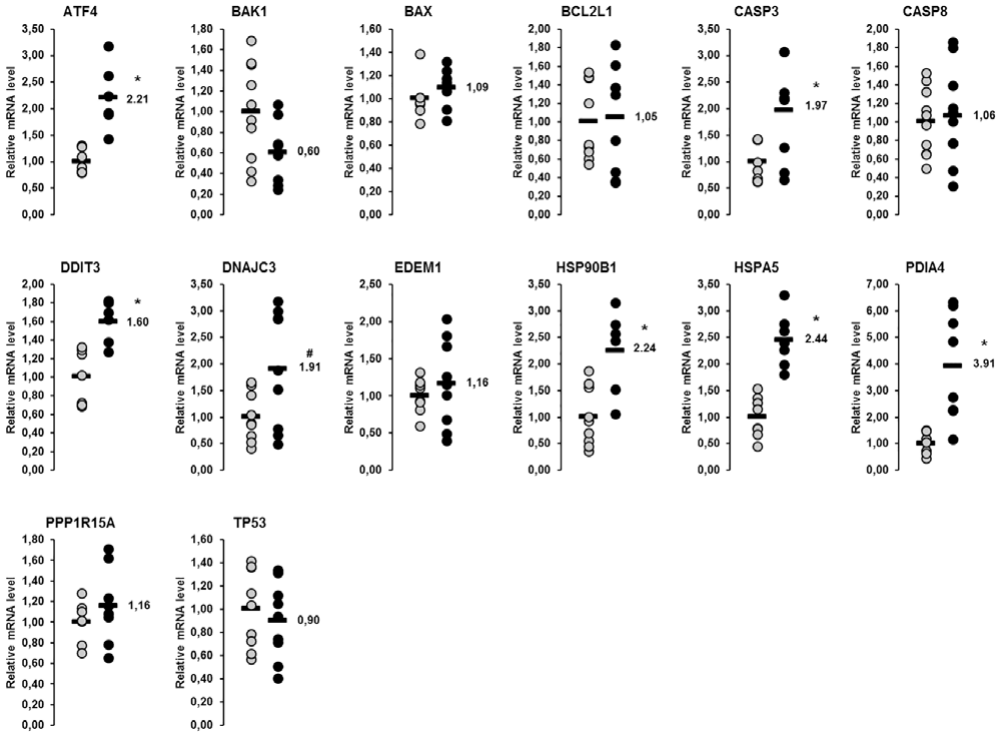
Effect of lactation on mRNA concentrations of genes involved in the UPR in the liver of sows. Relative mRNA concentrations of genes involved in the unfolded protein response (UPR) in the liver of lactating and non-lactating sows on day 20 of lactation. Filled circles (grey = non-lactating group, black = lactating group) represent individual data for each animal. Black lines represent means of individual data for each group (n = 6–10 sows per group). The mean of the non-lactating group is set to 1. The mean of the lactating group is expressed as fold of the non-lactating group and numerically indicated next to the black line. Superscript symbol indicates difference from non-lactating group (*p<0.05, ^#^p<0.1). Abbreviations: ATF4, activating transcription factor 4; BAK1, BCL2-antagonist/killer 1; BAX, BCL2-associated X protein; BCL2L1, BCL2-like 1; CASP, caspase, apoptosis-related cysteine peptidase; DDIT3, DNA-damage-inducible transcript 3; DNAJC3, DnaJ (Hsp40) homolog, subfamily C, member 3; EDEM1, ER degradation enhancer, mannosidase alpha-like 1; HSP90B1, heat shock protein 90kDa beta (Grp94), member 1; HSPA5, heat shock 70kDa protein 5 (glucose-regulated protein, 78kDa); PDIA4, protein disulfide isomerase family A, member 4; PPP1R15A, protein phosphatase 1, regulatory subunit 15A; TP53, tumor protein p53.

**Fig 2:**
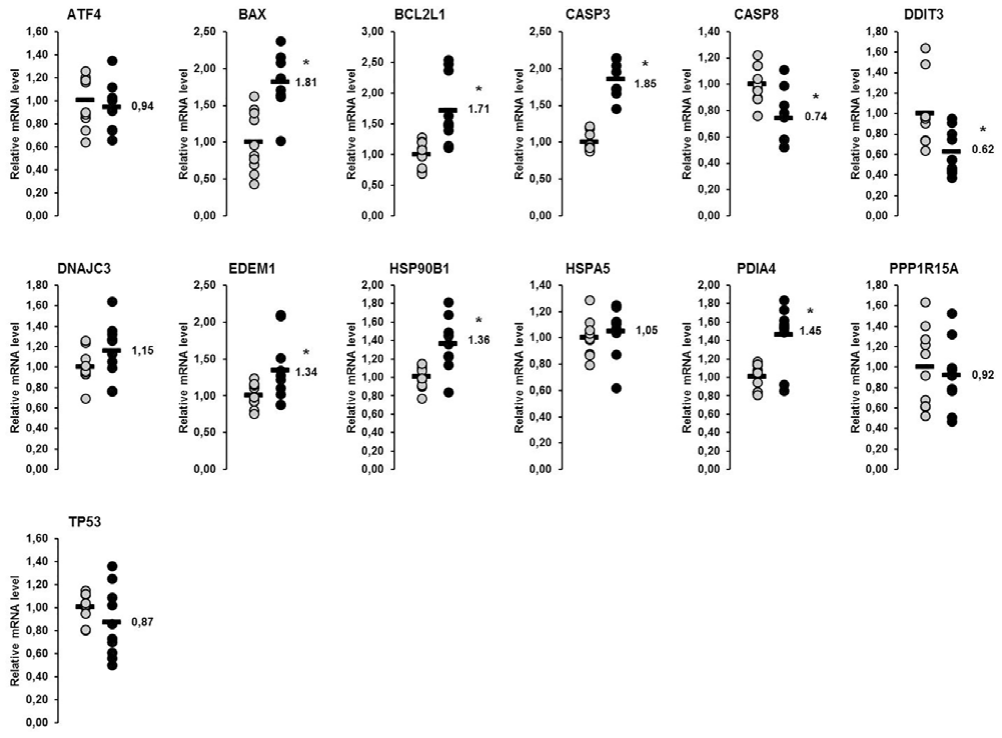
Effect of lactation on mRNA concentrations of genes involved in the UPR in the skeletal muscle of sows. Relative mRNA concentrations of genes involved in the UPR in the skeletal muscle of lactating and non-lactating sows on day 20 of lactation. Filled circles (grey = non-lactating group, black = lactating group) represent individual data for each animal. Black lines represent means of individual data for each group (n = 8–10 sows per group). The mean of the non-lactating group is set to 1. The mean of the lactating group is expressed as fold of the non-lactating group and numerically indicated next to the black line. Superscript symbol indicates difference from non-lactating group (*p<0.05). Abbreviations: ATF4, activating transcription factor 4; BAX, BCL2-associated X protein; BCL2L1, BCL2-like 1; CASP, caspase, apoptosis-related cysteine peptidase; DDIT3, DNA-damage-inducible transcript 3; DNAJC3, DnaJ (Hsp40) homolog, subfamily C, member 3; EDEM1, ER degradation enhancer, mannosidase alpha-like 1; HSP90B1, heat shock protein 90kDa beta (Grp94), member 1; HSPA5, heat shock 70kDa protein 5 (glucose-regulated protein, 78kDa); PDIA4, protein disulfide isomerase family A, member 4; PPP1R15A, protein phosphatase 1, regulatory subunit 15A; TP53, tumor protein p53.

In the liver, a total of 14 genes involved in the UPR were considered in this study. Amongst them, the relative mRNA concentrations of six genes (*ATF4*, *CASP3*, *DDIT3*, *HSP90B1*, *HSPA5*, *PDIA4*) were higher in lactating than in non-lactating sows (p<0.05), whereas that of one gene (*DNAJC3*) tended to be higher in the lactating than in the non-lactating sows (p<0.1; Fig 1). In skeletal muscle, 13 genes encoding proteins of the UPR were considered, from which the mRNA concentrations of six genes (*BAX*, *BCL2L1*, *CASP3*, *EDEM1*, *HSP90B1*, *PDIA4*) were higher and mRNA concentrations of two genes were lower (*CASP8*, *DDIT3*) in lactating than in non-lactating sows (p<0.05; Fig 2). The mRNA concentrations of the other genes investigated did not differ between the lactating and the non-lactating sows (Fig 2).

### Relative mRNA concentrations of genes involved in the NLRP3 inflammasome in the liver and skeletal muscle of lactating and non-lactating sows on day 20 of lactation

**Fig 3:**
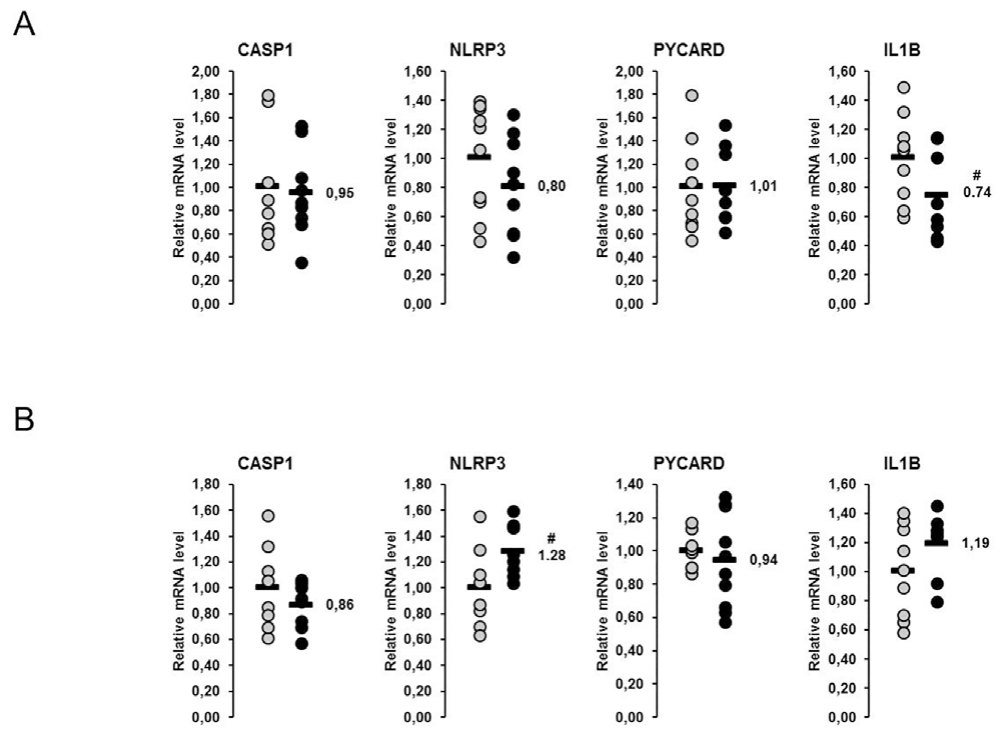
Effect of lactation on mRNA concentrations of genes involved in the NLRP3 inflammasome in the liver and skeletal muscle of sows. Relative mRNA concentrations of genes involved in the NOD-like receptor P3 (NLRP3) inflammasome the liver (A) and the skeletal muscle (B) of lactating and non-lactating sows on day 20 of lactation. Filled circles (grey = non-lactating group, black = lactating group) represent individual data for each animal. Black lines represent means of individual data for each group (n = 8–10 sows per group). The mean of the non-lactating group is set to 1. The mean of the lactating group is expressed as fold of the non-lactating group and numerically indicated next to the black line. Superscript symbol indicates difference from non-lactating group (^#^p<0.1). Abbreviations: CASP1, caspase 1, apoptosis-related cysteine peptidase; IL1B, interleukin 1, beta; NLRP3, NLR family, pyrin domain containing 3; PYCARD, PYD and CARD domain containing.

In both liver and skeletal muscle four genes involved in the NLRP3 inflammasome were considered (*CASP1*, *NLRP3*, *PYCARD*, *IL1B*). The relative mRNA concentrations of none of these genes in liver and skeletal muscle were higher in the lactating than in non-lactating sows (Fig 3A). The relative mRNA concentration of *IL1B* in the liver tended to be lower in lactating than in non-lactating sows (p<0.1; Fig 3A). In skeletal muscle, relative mRNA concentration of *NLRP3* tended to be higher in lactating than in non-lactating sows (p<0.1; Fig 3B).

### Relative protein concentrations of the ER stress sensor PERK and the ER stress targets eIF2α and IκB in the liver of lactating and non-lactating sows on day 20 of lactation

**Fig 4:**
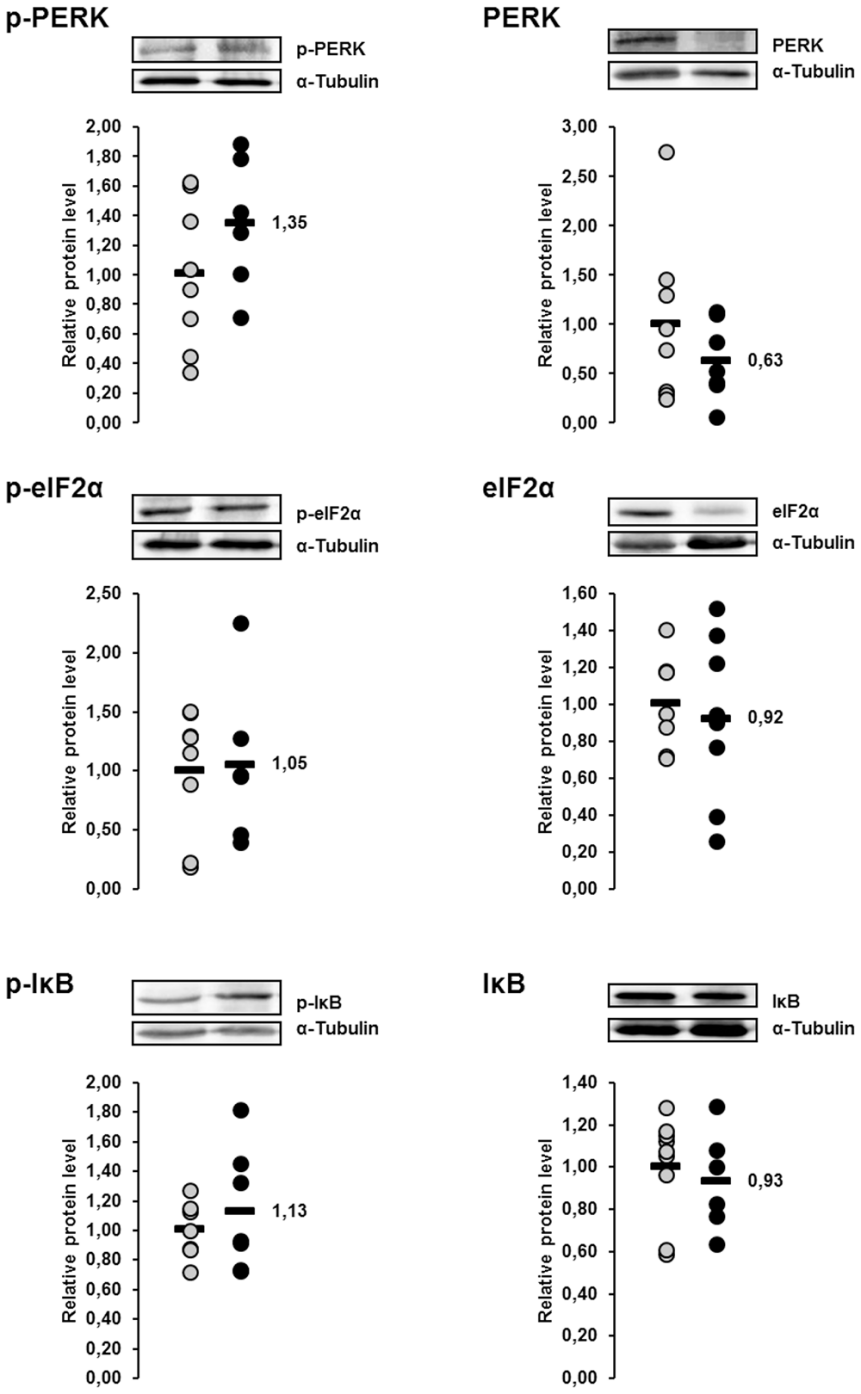
Effect of lactation on phosphorylation of PERK and PERK-mediated phosphorylation of eIF2α and IκB in the liver of sows. Relative protein concentrations of phosphorylated and total PKR-like ER kinase (PERK), eukaryotic translation initiation factor 2α (eIF2α) and inhibitor of κB (IκB) in the liver of lactating and non-lactating sows on day 20 of lactation. Representative immunoblots specific to phosphorylated and total PERK, eIF2α and IκB and α-Tubulin as internal control are shown for one animal per group; immunoblots for the other animals revealed similar results. Filled circles (grey = non-lactating group, black = lactating group) represent individual data for each animal from densitometric analysis. Black lines represent means of individual data for each group (n = 6–8 sows per group). The mean of the non-lactating group is set to 1. The mean of the lactating group is expressed as fold of the non-lactating group and numerically indicated next to the black line.

The protein concentrations of p-PERK, p-eIF2α and p-IκB in the liver were 35%, 5% and 13%, respectively, higher in lactating sows than in non-lactating sows, whereas the protein concentrations of total PERK, total eIF2α and total IκB in the liver were 38%, 8% and 7%, respectively, lower in lactating sows than in non-lactating sows (Fig 4). All of these effects were not statistically significant.

### Relative mRNA concentrations of NF-κB target genes in the liver and skeletal muscle of lactating sows with or without fish oil supplementation on day 20 of lactation

**Fig 5:**
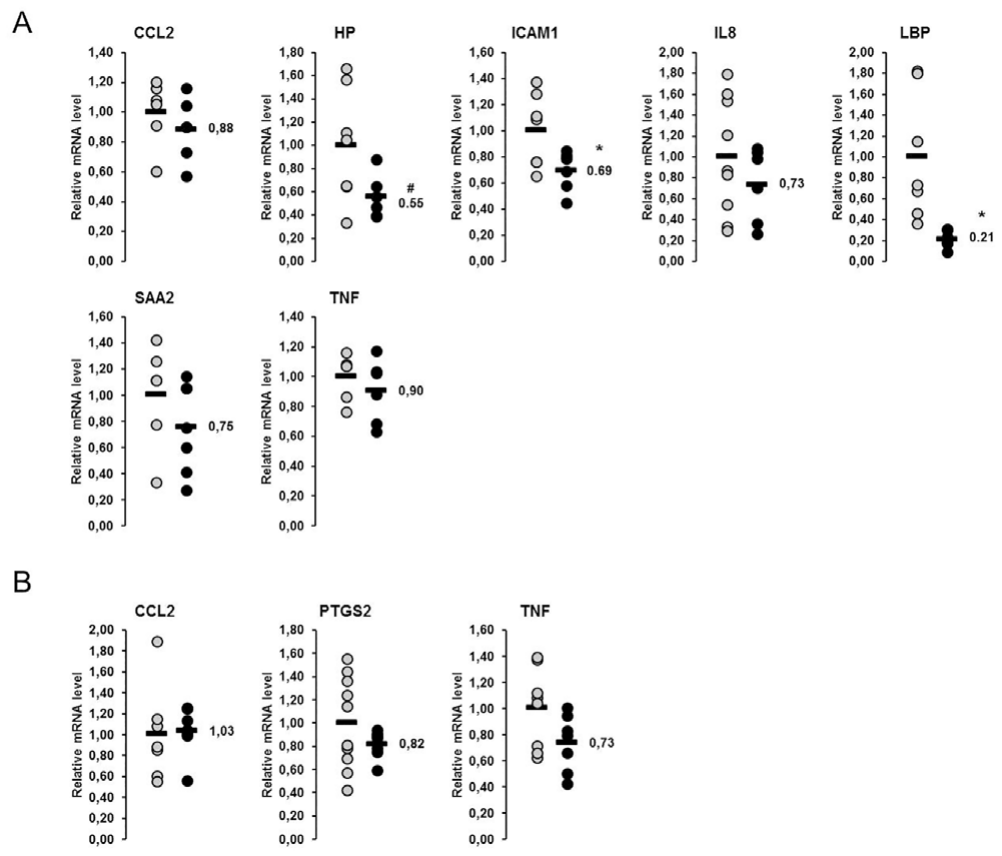
Effect of fish oil on mRNA concentrations of NF-κB target genes in the liver and skeletal muscle of lactating sows. Relative mRNA concentrations of nuclear factor κB (NF-κB) target genes in the liver (A) and the skeletal muscle (B) of lactating sows with or without fish oil supplementation on day 20 of lactation. Filled circles (grey = control group, black = fish oil group) represent individual data for each animal. Black lines represent means of individual data for each group (n = 6–10 sows per group). The mean of the control group is set to 1. The mean of the fish oil group is expressed as fold of the control group and numerically indicated next to the black line. Superscript symbol indicates difference from control group (*p<0.05, ^#^p<0.1). Abbreviations: CCL2, chemokine (C-C motif) ligand 2; HP, haptoglobin; ICAM1, intercellular adhesion molecule 1; IL8, interleukin 8; LBP, lipopolysaccharide binding protein; PTGS2, prostaglandin-endoperoxide synthase 2; SAA2, serum amyloid A2; TNF, tumor necrosis factor.

In the liver, relative mRNA concentrations of the seven NF-κB target genes determined were reduced by 10–79% in comparison to the control group; significant differences (p<0.05) between the two groups of sows were observed for the mRNA concentrations of *LBP* and *ICAM1* (Fig 5A). The mRNA concentration of *HP* in the liver tended to be reduced in the fish oil group (p<0.1; Fig 5A). In skeletal muscle, the relative mRNA concentrations of three NF-κB target genes were considered, from which none was different between sows of the fish oil group and the control group (Fig 5B).

### Relative mRNA concentrations of genes involved in the UPR in the liver and skeletal muscle of lactating sows with or without fish oil supplementation on day 20 of lactation

**Fig 6:**
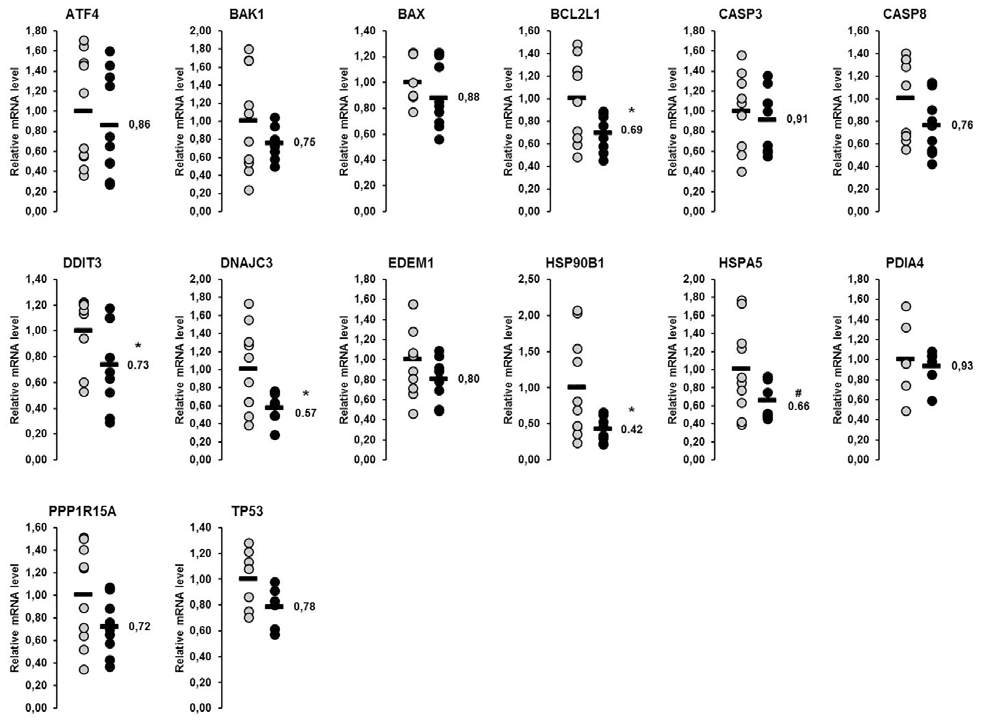
Effect of fish oil on mRNA concentrations of genes involved in the UPR in the liver of lactating sows. Relative mRNA concentrations of genes involved in the unfolded protein response (UPR) in the liver of lactating sows with or without fish oil supplementation on day 20 of lactation. Filled circles (grey = control group, black = fish oil group) represent individual data for each animal. Black lines represent means of individual data for each group (n = 6–10 sows per group). The mean of the control group is set to 1. The mean of the fish oil group is expressed as fold of the control group and numerically indicated next to the black line. Superscript symbol indicates difference from control group (*p<0.05, ^#^p<0.1). Abbreviations: ATF4, activating transcription factor 4; BAK1, BCL2-antagonist/killer 1; BAX, BCL2-associated X protein; BCL2L1, BCL2-like 1; CASP, caspase, apoptosis-related cysteine peptidase; DDIT3, DNA-damage-inducible transcript 3; DNAJC3, DnaJ (Hsp40) homolog, subfamily C, member 3; EDEM1, ER degradation enhancer, mannosidase alpha-like 1; HSP90B1, heat shock protein 90kDa beta (Grp94), member 1; HSPA5, heat shock 70kDa protein 5 (glucose-regulated protein, 78kDa); PDIA4, protein disulfide isomerase family A, member 4; PPP1R15A, protein phosphatase 1, regulatory subunit 15A; TP53, tumor protein p53.

**Fig 7:**
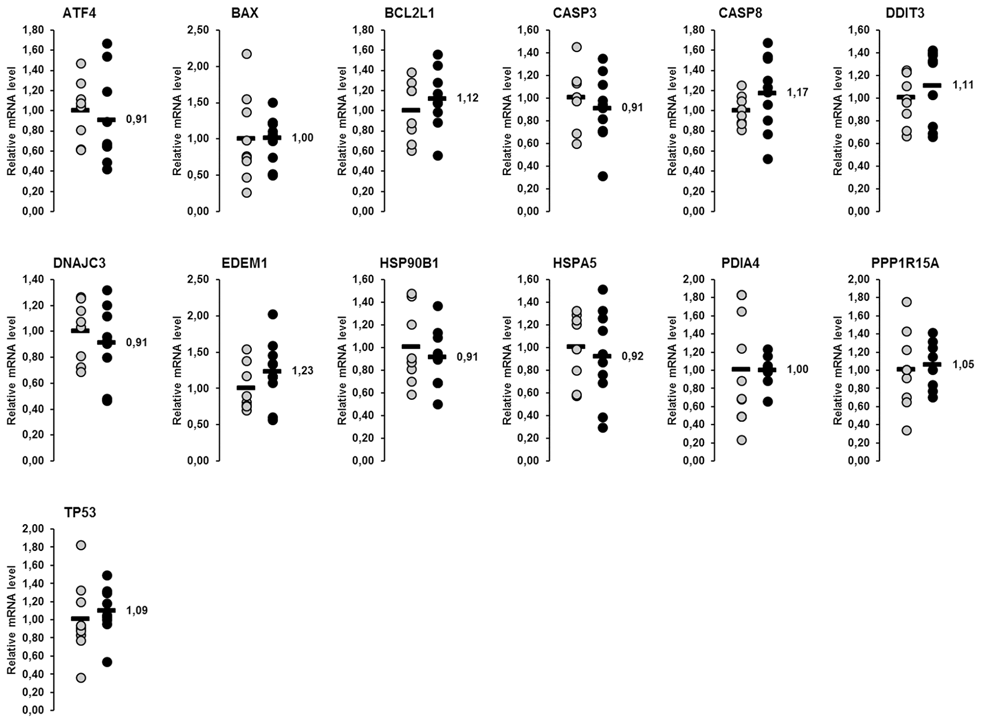
Effect of fish oil on mRNA concentrations of genes involved in the UPR in the skeletal muscle of lactating sows. Relative mRNA concentrations of genes involved in the unfolded protein response (UPR) in the skeletal muscle of lactating sows with or without fish oil supplementation on day 20 of lactation. Filled circles (grey = control group, black = fish oil group) represent individual data for each animal. Black lines represent means of individual data for each group (n = 7–10 sows per group). The mean of the control group is set to 1. The mean of the fish oil group is expressed as fold of the control group and numerically indicated next to the black line. Abbreviations: ATF4, activating transcription factor 4; BAX, BCL2-associated X protein; BCL2L1, BCL2-like 1; CASP, caspase, apoptosis-related cysteine peptidase; DDIT3, DNA-damage-inducible transcript 3; DNAJC3, DnaJ (Hsp40) homolog, subfamily C, member 3; EDEM1, ER degradation enhancer, mannosidase alpha-like 1; HSP90B1, heat shock protein 90kDa beta (Grp94), member 1; HSPA5, heat shock 70kDa protein 5 (glucose-regulated protein, 78kDa); PDIA4, protein disulfide isomerase family A, member 4; PPP1R15A, protein phosphatase 1, regulatory subunit 15A; TP53, tumor protein p53.

In the liver, the mRNA concentrations of the 14 genes involved in the UPR considered were reduced by 9–58% in the fish oil group compared to the control group (Fig 6). The reduction of mRNA concentrations of these genes was significant for four genes: *BCL2L1*, *DDIT3*, *DNAJC3* and *HSP90B1* (p<0.05; Fig 6). The relative mRNA concentration of *HSPA5* tended to be lower in the fish oil group compared to the control group (p<0.1; Fig 6). In skeletal muscle, the mRNA concentrations of none of the 13 UPR target genes investigated were different between sows of the fish oil group and the control group (Fig 7).

### Relative mRNA concentrations of Nrf2 target genes in the liver and skeletal muscle of lactating sows with or without fish oil supplementation on day 20 of lactation

**Fig 8:**
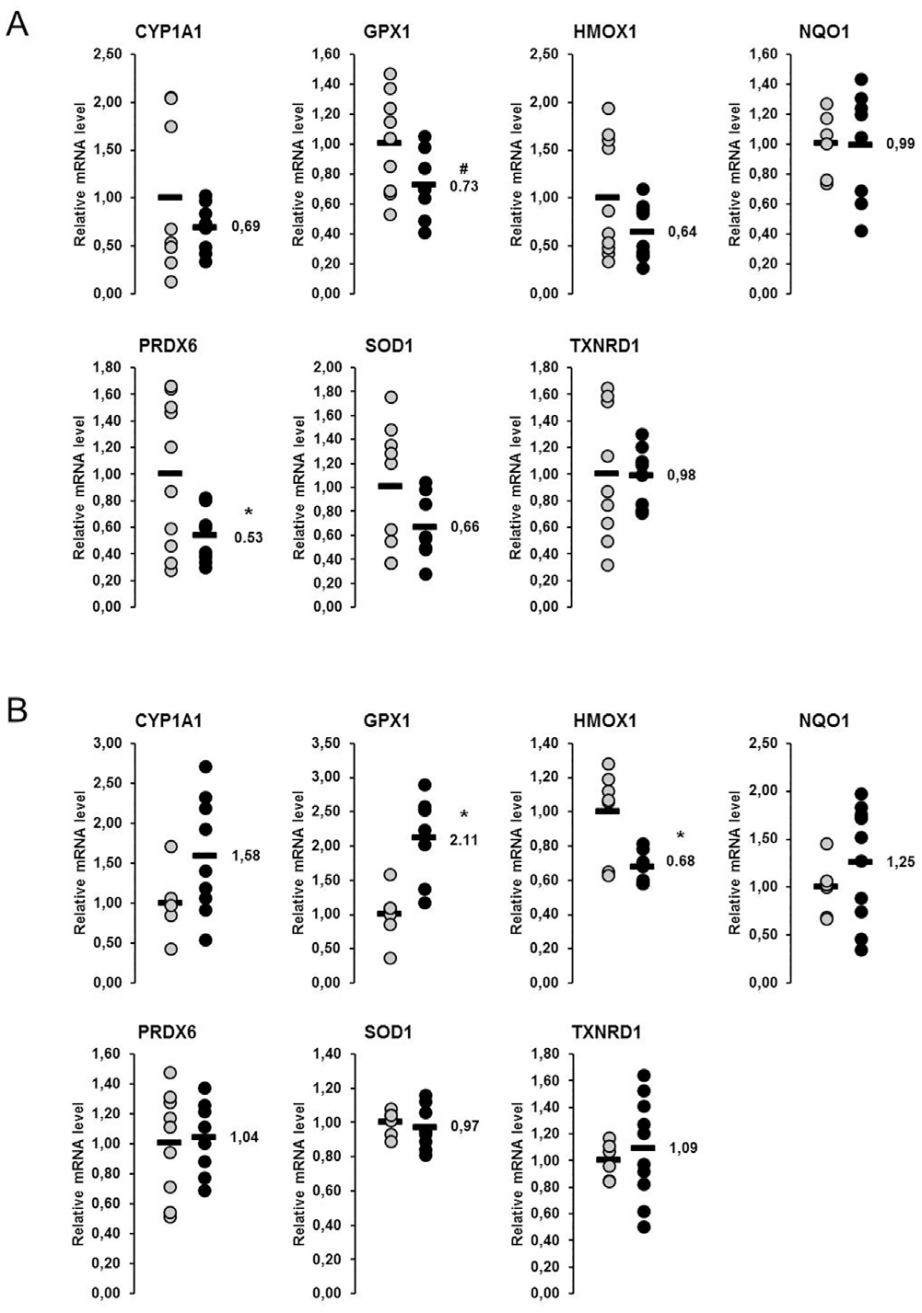
Effect of fish oil on mRNA concentrations of Nrf2 target genes in the liver and skeletal muscle of lactating sows. Relative mRNA concentrations of nuclear factor E2-related factor 2 (Nrf2) target genes in the liver (A) and the skeletal muscle (B) of lactating sows with or without fish oil supplementation on day 20 of lactation. Filled circles (grey = control group, black = fish oil group) represent individual data for each animal. Black lines represent means of individual data for each group (n = 6–10 sows per group). The mean of the control group is set to 1. The mean of the fish oil group is expressed as fold of the control group and numerically indicated next to the black line. Superscript symbol indicates difference from control group (*p<0.05, ^#^p<0.1). Abbreviations: CYP1A1, cytochrome P450, family 1, subfamily A, polypeptide 1; GPX1, glutathione peroxidase 1; HMOX1, heme oxygenase 1; NQO1, NAD(P)H dehydrogenase, quinone 1; PRDX6, peroxiredoxin 6; SOD1, superoxide dismutase 1, soluble; TXNRD1, thioredoxin reductase 1.

In the liver, relative mRNA concentrations of the seven Nrf2 target genes considered were reduced by 1–47% in the fish oil group compared to the control group (Fig 8A); a significant reduction (p<0.05) was observed for *PRDX6*, whereas the reduction of *GPX1* tended to be significant (p<0.1). In skeletal muscle, the relative mRNA concentrations of the same seven Nrf2 target genes were considered. Five of them were not different between the fish oil group and the control group, whereas one (*GPX1*) was increased (p<0.05) and one (*HMOX1*) was decreased (p<0.05), respectively, in the fish oil group compared to the control group (Fig 8B).

### Relative mRNA concentrations of genes involved in the NLRP3 inflammasome in the liver and skeletal muscle of lactating sows with or without fish oil supplementation on day 20 of lactation

**Fig 9:**
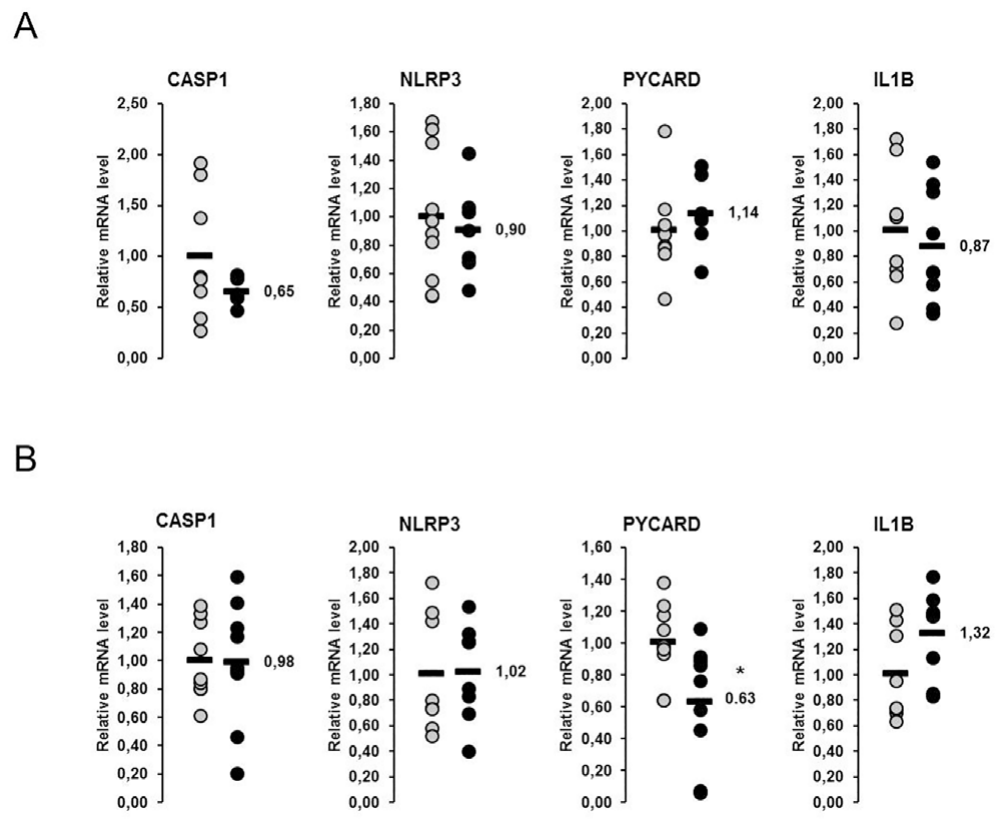
Effect of fish oil on mRNA concentrations of genes involved in the NLRP3 inflammasome in the liver and skeletal muscle of lactating sows. Relative mRNA concentrations genes involved in the NOD-like receptor P3 (NLRP3) inflammasome in the liver (A) and the skeletal muscle (B) of lactating sows with or without fish oil supplementation on day 20 of lactation. Filled circles (grey = control group, black = fish oil group) represent individual data for each animal. Black lines represent means of individual data for each group (n = 6–10 sows per group). The mean of the control group is set to 1. The mean of the fish oil group is expressed as fold of the control group and numerically indicated next to the black line. Superscript symbol indicates difference from control group (*p<0.05). Abbreviations: CASP1, caspase 1, apoptosis-related cysteine peptidase; IL1B, interleukin 1, beta; NLRP3, NLR family, pyrin domain containing 3; PYCARD, PYD and CARD domain containing.

The relative mRNA concentrations of four genes involved in the NLRP3 inflammasome (*CASP1*, *NLRP3*, *PYCARD*, *IL1B*) in the liver did not differ between the fish oil group and the control group (Fig 9A). In skeletal muscle the relative mRNA concentration of *PYCARD* was reduced in the fish oil group compared to the control group (p<0.05; Fig 9B), whereas the other genes (*CASP1*, *NLRP3*, *IL1B*) were not different between groups.

### Relative protein concentrations of the ER stress sensor PERK and the ER stress targets eIF2α and IκB in the liver of lactating sows with or without fish oil supplementation on day 20 of lactation

**Fig 10:**
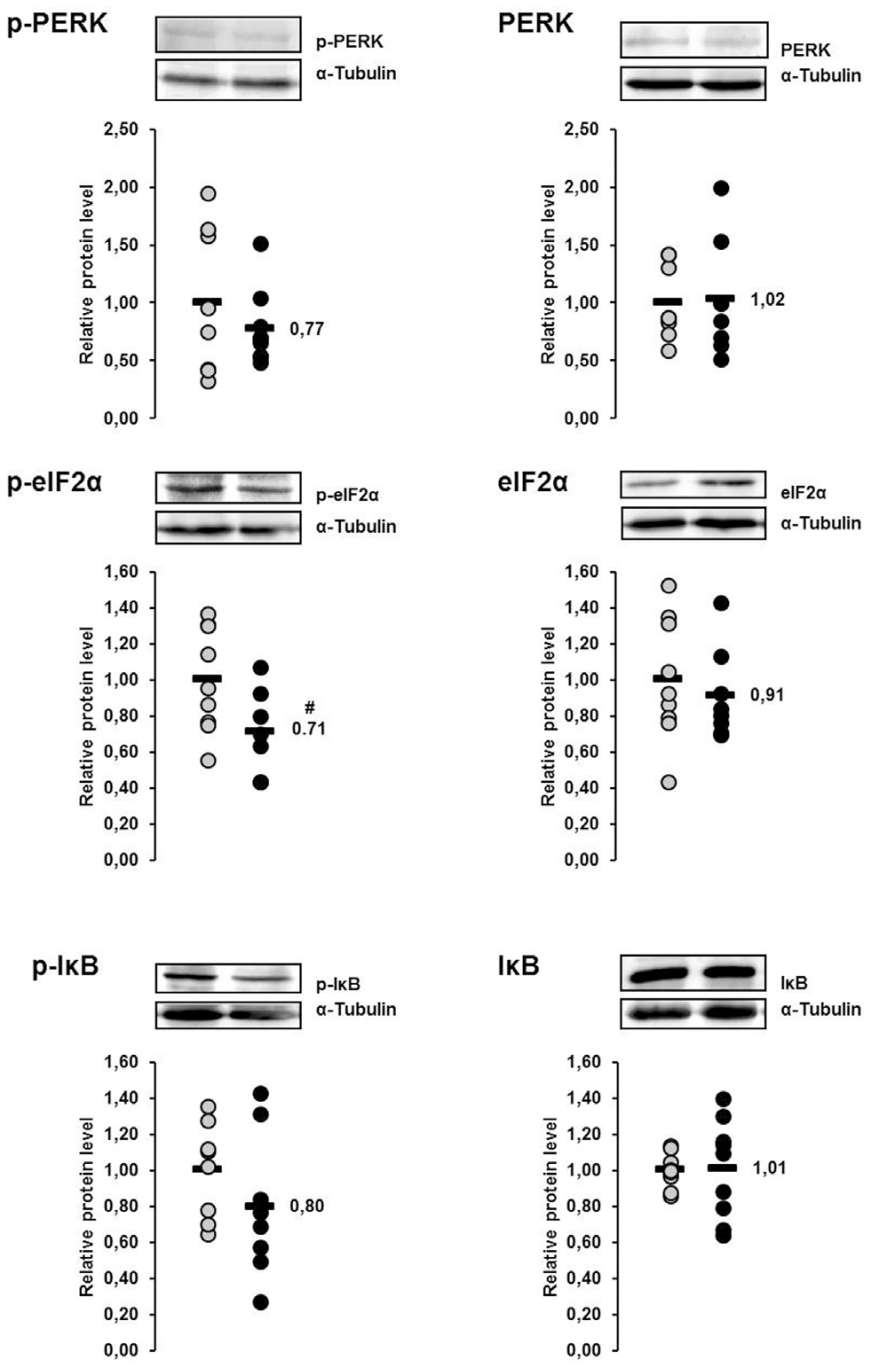
Effect of fish oil on phosphorylation of PERK and PERK-mediated phosphorylation of eIF2α and IκB in the liver of lactating sows. Relative protein concentrations of phosphorylated and total (PERK), eukaryotic translation initiation factor 2α (eIF2α) and inhibitor of κB (IκB) in the liver of lactating sows with or without fish oil supplementation on day 20 of lactation. Representative immunoblots specific to phosphorylated and total PERK, eIF2α and IκB and α-Tubulin as internal control are shown for one animal per group; immunoblots for the other animals revealed similar results. Filled circles (grey = control group, black = fish oil group) represent individual data for each animal from densitometric analysis. Black lines represent means of individual data for each group (n = 7–9 sows per group). The mean of the control group is set to 1. The mean of the fish oil group is expressed as fold of the control group and numerically indicated next to the black line. Superscript symbol indicates difference from control group (^#^p<0.1).

The protein concentrations of p-PERK, p-eIF2α and p-IκB in the liver were 23%, 29% and 20%, respectively, lower in sows of the fish oil group than in those of the control group (Fig 10). The p-value of the effect on p-eIF2α was <0.1, whereas the other effects were not statistically significant. The protein concentrations of total PERK, total eIF2α and total IκB in the liver did not differ between the two groups (Fig 10).

### Plasma concentrations of HP und CRP in lactating sows with or without fish oil supplementation on day 20 of lactation

The plasma concentrations of HP and CRP were 10–20% lower in sows of the fish oil group than in those of the control group (HP: 2.33 ± 0.59 vs. 2.67 ± 0.81 mg/mL; CRP: 197 ± 125 vs. 324 ± 221 μg/mL), but these differences were not statistically significant.

## Discussion

We have recently reported that the liver of lactating sows develops typical signs of an inflammatory condition, such as activation of NF-κB and up-regulation of genes encoding APPs, as a consequence of the metabolic and physiologic adaptations occurring during the transition from pregnancy to lactation [12]. Hepatic inflammation and the associated acute phase response are critical because it impairs performance of farm animals and it results in systemically elevated levels of inflammatory mediators (APPs, cytokines) and ROS. Both, inflammatory mediators and ROS are well known stimulators of ER stress that is known to induce the adaptive UPR [17]. One key finding of the present study is that ER stress-induced UPR occurs also in the liver of sows during lactation. This was evident from the up-regulation of a number of genes regulated by the UPR including the ER chaperones *HSP90B1* and *HSPA5*, the protein disulfide isomerase *PDIA4*, the key regulator of ER-stress induced apoptosis *DDIT3*, the apoptotic protein *CASP3*, and *ATF4*, which is the main regulator of DDIT3 and ERAD components. All these genes are downstream target genes of the three ER stress transducers inositol requiring 1 (IRE1), PKR-like ER kinase (PERK) and activating factor 6 (ATF6), and, thus, these genes are considered reliable markers of ER stress [40]. Moreover, we found that phosphorylation of the ER stress transducer PERK and phosphorylation of the ER stress targets eIF2α and IκB were increased, at least numerically, in the liver of lactating sows. Collectively, these results strongly suggest that, like in high-yielding dairy cows [27], ER stress and the induction of the UPR occur in the liver of lactating sows. Like in the liver, at least some of the UPR-regulated genes considered were found to be up-regulated in skeletal muscle of lactating sows, which indicates that the occurrence of ER stress-induced UPR during lactation is not restricted to the liver but is present also in non-hepatic tissues.

A second main finding of the present study is that feeding fish oil as a source of anti-inflammatory n-3 PUFA inhibits the ER stress-induced UPR in the liver of lactating sows, as evidenced from down-regulation of UPR target genes and decreased phosphorylation of PERK, eIF2α and IκB. In addition, we observed that the mRNA levels of NF-κB regulated genes encoding inflammatory mediators, like APPs, cytokines, chemokines and adhesion molecules, in the liver of lactating sows were reduced, at least numerically, in the fish oil group compared to the control group. Considering the down-regulation of all genes investigated in the liver in the fish oil group it has to be pointed out that this is a specific biological effect of fish oil, because we have recently demonstrated that fish oil is also able to cause an up-regulation of genes, like lipoprotein lipase, cytochrome P450 4A24, of certain metabolic pathways in the liver of sows [35]. In line with the inhibition of inflammatory gene expression, we found 13 to 39% decreased plasma levels of HP and CRP, respectively. HP and CRP are two of the main positive APPs produced in the liver of pigs within the acute phase response [41–44], and both APPs have been shown to be elevated in plasma of sows one week after farrowing compared to late pregnancy [14]. Although the effect of fish oil on the plasma levels of APPs was not significant due to the large biological variation between individual sows, which has been reported also from others [45], our observation indicates that fish oil is able to attenuate the pro-inflammatory process associated with lactation. Since inflammatory mediators are important signals for the induction of ER stress, our findings suggest that fish oil inhibits the ER stress-induced UPR by attenuating the inflammatory condition in the liver of lactating sows. Inhibition of hepatic NF-κB by fish oil in the liver probably also provides the molecular basis for the observation from Papadopoulos et al. [33] that a diet with a low n-6:n-3 PUFA ratio reduces plasma levels of the APP SAA in lactating sows. The inhibitory effect of n-3 PUFA on NF-κB has long been known and is explained by PPARα-mediated transrepression of NF-κB due the ability of n-3 PUFA to bind to and activate PPARα [46].

A further interesting finding of the present study is that feeding of fish oil decreases the mRNA levels of Nrf2-regulated genes in the liver of lactating sows. This finding is also indicative of inhibition of ER stress, because activation of the cytoprotective Nrf2 pathway has been shown to be the consequence of ER stress and to be mediated through the ER stress inducer PERK [24]. Thus, our recent observation that the Nrf2 pathway is activated in the liver of lactating sows compared to non-lactating sows [12] is a further indirect evidence for the occurrence of ER stress in the liver of sows during lactation. Like in lactating sows, activation of Nrf2 was found recently in the liver of high-yielding dairy cows during early lactation [47]. In lactating cows, this effect has been interpreted as a compensatory means to protect the liver against ROS- and inflammation-induced damage [47], because Nrf2 controls the transcription of various antioxidative and cytoprotective proteins. Thus, the physiologic meaning of Nrf2 activation in the liver of sows during lactation might be the same as in dairy cows.

In contrast to the liver, feeding fish oil failed to inhibit the ER stress-induced UPR in skeletal muscle of lactating sows as shown by unaltered mRNA levels of UPR-regulated genes. Similarly, feeding fish oil largely did not reduce the expression of NF-κB and Nrf2 target genes in skeletal muscle, suggesting that fish oil did not exert an anti-inflammatory and cytoprotective action in skeletal muscle of the lactating sows. Since a pro-inflammatory environment induces ER stress, the observation that fish oil did not attenuate skeletal muscle expression of pro-inflammatory NF-κB target genes is likely responsible for the lack of inhibition of ER stress-induced UPR in skeletal muscle of the lactating sows. We have no true explanation for the lack of anti-inflammatory effect of fish oil in skeletal muscle, but it may be explained by a lower availability of n-3 PUFA in skeletal muscle than in the liver. It is well known that one important mechanism explaining the anti-inflammatory effects of n-3 PUFA from fish oil, such as EPA and DHA, is that they compete with arachidonic acid in the membrane phospholipids for cyclooxygenase and lipoxygenase, with the consequent production of less potent inflammatory eicosanoids and of anti-inflammatory mediators such as resolvins. Although we did not analyse the proportions of n-3 PUFA and arachidonic acid in skeletal muscle and liver lipids due to the limited amount of liver and skeletal muscle biopsy samples, we postulate that the dietary n-3 PUFA taken up from the fish oil were incorporated at greater levels into the liver lipids than into the muscle lipids. This assumption is based on several studies in pigs and rats showing that dietary n-3 PUFA are incorporated to a greater extent into liver lipids than into skeletal muscle or adipose tissue lipids [48,49].

In the present study, we also considered the NLRP3 inflammasome pathway, a pro-inflammatory signaling pathway which has not yet been investigated in lactating sows. This pathway is known to be activated by “danger” signals like saturated fatty acids, but also ROS and pathogen-associated molecular patterns, such as lipopolysaccharides, microbial proteins and double-stranded ribonucleic acids, and to mediate the release of pro-inflammatory cytokines, such as IL-1B and IL-18 [34]. In contrast to the other stress pathways (UPR, NF-κB, Nrf2) considered, we found no evidence for an activation of the NLRP3 inflammasome pathway in neither liver nor skeletal muscle of sows during lactation. This was demonstrated by unaltered mRNA concentrations of four NLRP3 inflammasome-related genes (*CASP1*, *NLRP3*, *PYCARD*, *IL1B*) in tissues of lactating and non-lactating sows. In addition, administration of fish oil failed to reduce the expression of most of the NLRP3 inflammasome-related genes in liver and skeletal muscle of lactating sows, although it has been reported in the literature that n-3 PUFA are able to inhibit NLRP3 inflammasome activation, at least in human THP-1 cells [50]. At the moment, we have no explanation for the lack of effect of lactation and fish oil treatment on the NLRP3 inflammasome pathway in sows, but it is possible that further genes involved in this pathway have to be considered to obtain a more meaningful picture about regulation of this pathway by lactation and fish oil in sows. Further investigations on this issue are warranted in future studies.

Our study has one limitation: When compared to the typical litter size of high-yielding genotypes in modern pig production, the litter size in the second study (8 piglets/sow) was quite small. This indicates that the energy requirement for milk production and consequently the lactation-induced metabolic stress and the induction of pro-inflammatory and ER stress signalling pathways was lower in the second than in the first study. Indeed, we have recently reported that the lactating sows of the first study were in a strong negative energy balance of approximately -35 MJ ME/day suggesting that the increase of feed intake during lactation was not sufficient to fully compensate the increased energy requirement for milk production [13]. In contrast, the lactating sows of both the control and the fish oil group in the second study had only a slightly negative energy balance of approximately -5 MJ ME/day [35]. Accordingly, the obviously lower metabolic stress in sows of the second study may explain that the down-regulation of inflammatory and ER stress-related genes in the liver of the fish oil group was only moderate. Nevertheless, the observation that fish oil was able to inhibit the expression of all of these genes in sows that experienced only moderate stress indicates that fish oil is an efficient dietary approach to combat lactation-induced metabolic and inflammatory stress in sows. Thus, future studies are warranted investigating the efficacy of dietary fish oil in high-yielding genotypes with markedly greater litter sizes of 15 and more piglets. In addition, such studies should include several time points during lactation for tissue sample collection in order to consider possible dynamic changes of the inflammatory and ER stress response in sows during lactation.

## Conclusion

Our study shows for the first time that, like in dairy cows, ER stress-induced UPR is present in the liver and skeletal muscle of sows during lactation, and dietary fish oil is able to inhibit, at least in the liver, ER stress-induced UPR and inflammatory and stress signaling pathways, which are involved in the induction of ER stress. The occurrence of ER stress in the liver during lactation indicates that the metabolic and physiologic changes occurring during the transition from pregnancy to lactation represent cellular stress that might be detrimental to health and performance of sows. At least in dairy cows it has been suggested that the ER stress-induced UPR contributes to the pathophysiologic conditions commonly observed in the liver of periparturient cows [27], such as fatty liver and ketosis, which are considered critical with regard to milk and reproductive performance. Although little is known about the occurrence of liver-associated diseases and its relevance for milk and reproductive performance in lactating sows, it is assumed that feeding fish oil is a useful dietary strategy to improve health and performance of lactating sows.

## References

[1] Aiello RJ, Kenna TM, Herbein JH. Hepatic gluconeogenic and ketogenic interrelationships in the lactating cow. J Dairy Sci. 1984; 67: 1707–1715. 6480960 10.3168/jds.S0022-0302(84)81496-4

[2] Collier RJ, McNamara JP, Wallace CR, Dehoff MH. A review of endocrine regulation of metabolism during lactation. J Anim Sci. 1984; 59: 498–510. 6090379 10.2527/jas1984.592498x

[3] Trottier NL, Easter RA. Dietary and plasma branched-chain amino acids in relation to tryptophan: effect on voluntary feed intake and lactation metabolism in the primiparous sow. J Anim Sci. 1995; 73: 1086–1092. 7628952 10.2527/1995.7341086x

[4] Trayhurn P, Douglas JB, McGuckin MM. Brown adipose tissue thermogenesis is 'suppressed' during lactation in mice. Nature. 1982; 298: 59–60. 6283369 10.1038/298059a0

[5] Williamson DH. Fuel supply to brown adipose-tissue. Biochem Soc Trans. 1986; 14: 225–227. 3709945 10.1042/bst0140225

[6] Dewey KG. Energy and protein requirements during lactation. Annu Rev Nutr. 1997; 17: 19–36. 9240917 10.1146/annurev.nutr.17.1.19

[7] Smith MS, Grove KL. Integration of the regulation of reproductive function and energy balance: lactation as a model. Front Neuroendocrinol. 2002; 23: 225–256. 12127305 10.1016/s0091-3022(02)00002-x

[8] Gutgesell A, Ringseis R, Brandsch C, Stangl GI, Hirche F, Eder K. Peroxisome proliferator-activated receptor α and enzymes of carnitine biosynthesis in the liver are down-regulated during lactation in rats. Metabolism. 2009; 58: 226–232. 10.1016/j.metabol.2008.09.018 19154956

[9] Gutgesell A, Ringseis R, Schmidt E, Brandsch C, Stangl GI, Eder K. Downregulation of peroxisome proliferator-activated receptor α and its coactivators in liver and skeletal muscle mediates the metabolic adaptations during lactation in mice. J Mol Endocrinol. 2009; 43: 241–250. 10.1677/JME-09-0064 19578095

[10] Drackley JK. Biology of dairy cows during the transition period: The final frontier? J Dairy Sci. 1999; 82: 2259–2273. 10575597 10.3168/jds.s0022-0302(99)75474-3

[11] Katoh N. Relevance of apolipoproteins in the development of fatty liver and fatty liver-related peripartum diseases in dairy cows. J Vet Med Sci. 2002; 64: 293–307. 12014573 10.1292/jvms.64.293

[12] Rosenbaum S, Ringseis R, Hillen S, Becker S, Erhardt G, Reiner G, et al The stress signalling pathway nuclear factor E2-related factor 2 is activated in the liver of sows during lactation. Acta Vet Scand. 2012; 54: 59 10.1186/1751-0147-54-59 23039904 PMC3502514

[13] Rosenbaum S, Ringseis R, Hillen S, Becker S, Erhardt G, Reiner G, et al Genome-wide transcript profiling indicates induction of energy-generating pathways and an adaptive immune response in the liver of sows during lactation. Comp Biochem Physiol Part D Genomics Proteomics. 2012; 7: 370–381. 10.1016/j.cbd.2012.09.001 23031603

[14] Kovac G, Tothova CS, Nagy O, Seidel H. Acute phase proteins during the reproductive cycle of sows. Acta Vet. 2008; 58: 459–466.

[15] Murata H, Shimada N, Yoshioka M. Current research on acute phase proteins in veterinary diagnosis: an overview. Vet J. 2004; 168: 28–40. 15158206 10.1016/S1090-0233(03)00119-9

[16] Bossaert P, Trevisi E, Opsomer G, Bertoni G, De Vliegher S, Leroy JL. The association between indicators of inflammation and liver variables during the transition period in high-yielding dairy cows: An observational study. Vet J. 2012; 192: 222–225. 10.1016/j.tvjl.2011.06.004 21742524

[17] Cnop M, Foufelle F, Velloso LA. Endoplasmic reticulum stress, obesity and diabetes. Trends Mol Med. 2012; 18: 59–68. 10.1016/j.molmed.2011.07.010 21889406

[18] Fu SN, Watkins SM, Hotamisligil GS. The role of endoplasmic reticulum in hepatic lipid homeostasis and stress signaling. Cell Metab. 2012; 15: 623–634. 10.1016/j.cmet.2012.03.007 22560215

[19] Marciniak SJ, Ron D. Endoplasmic reticulum stress signaling in disease. Physiol Rev. 2006; 86: 1133–1149. 17015486 10.1152/physrev.00015.2006

[20] Ron D, Walter P. Signal integration in the endoplasmic reticulum unfolded protein response. Nature Rev Mol Cell Biol. 2007; 8: 519–529. 17565364 10.1038/nrm2199

[21] Zhang KZ, Kaufman RJ. From endoplasmic-reticulum stress to the inflammatory response. Nature. 2008; 454: 455–462. 10.1038/nature07203 18650916 PMC2727659

[22] Lee JS, Zheng Z, Mendez R, Ha SW, Xie YM, Zhang K. Pharmacologic ER stress induces non-alcoholic steatohepatitis in an animal model. Toxicol Lett. 2012; 211: 29–38. 10.1016/j.toxlet.2012.02.017 22414386 PMC3334434

[23] Schaap FG, Kremer AE, Lamers WH, Jansen PLM, Gaemers IC. Fibroblast growth factor 21 is induced by endoplasmic reticulum stress. Biochimie. 2013; 95: 692–699. 10.1016/j.biochi.2012.10.019 23123503

[24] Cullinan SB, Zhang D, Hannink M, Arvisais E, Kaufman RJ, Diehl JA. Nrf2 is a direct PERK substrate and effector of PERK-dependent cell survival. Mol Cell Biol. 2003; 23: 7198–7209. 14517290 10.1128/MCB.23.20.7198-7209.2003 PMC230321

[25] Breckenridge DG, Germain M, Mathai JP, Nguyen M, Shore GC. Regulation of apoptosis by endoplasmic reticulum pathways. Oncogene. 2003; 22: 8608–8618. 14634622 10.1038/sj.onc.1207108

[26] Rutkowski DT, Kaufman RJ. A trip to the ER: coping with stress. Trends Cell Biol. 2004; 14: 20–28. 14729177 10.1016/j.tcb.2003.11.001

[27] Gessner DK, Schlegel G, Ringseis R, Schwarz FJ, Eder K. Up-regulation of endoplasmic reticulum stress induced genes of the unfolded protein response in the liver of periparturient dairy cows. BMC Vet Res. 2014; 10. 10.1186/1746-6148-10-46 PMC3936700 24555446

[28] Myhrstad MCW, Retterstol K, Telle-Hansen VH, Ottestad I, Halvorsen B, Holven KB, et al Effect of marine n-3 fatty acids on circulating inflammatory markers in healthy subjects and subjects with cardiovascular risk factors. Inflamm Res. 2011; 60: 309–319. 10.1007/s00011-010-0302-5 21229287 PMC3058501

[29] Miles EA, Calder PC. Influence of marine n-3 polyunsaturated fatty acids on immune function and a systematic review of their effects on clinical outcomes in rheumatoid arthritis. Br J Nutr. 2012; 107: S171–S184. 10.1017/S0007114512001560 22591891

[30] Rooke JA, Sinclair AG, Edwards SA. Feeding tuna oil to the sow at different times during pregnancy has different effects on piglet long-chain polyunsaturated fatty acid composition at birth and subsequent growth. Br J Nutr. 2001; 86: 21–30. 11432761 10.1079/bjn2001363

[31] Rooke JA, Sinclair AG, Ewen M. Changes in piglet tissue composition at birth in response to increasing maternal intake of long-chain n-3 polyunsaturated fatty acids are non-linear. Br J Nutr. 2001; 86: 461–470. 11591233 10.1079/bjn2001422

[32] Smits RJ, Luxford BG, Mitchell M, Nottle MB. Sow litter size is increased in the subsequent parity when lactating sows are fed diets containing n-3 fatty acids from fish oil. J Animal Sci. 2011; 89: 2731–2738. 10.2527/jas.2010-3593 21610255

[33] Papadopoulos GA, Maes DGD, Van Weyenberg S, van Kempen TATG, Buyse J, Janssen GP. Peripartal feeding strategy with different n-6:n-3 ratios in sows: effects on sows' performance, inflammatory and periparturient metabolic parameters. Br J Nutr. 2009; 101: 348–357. 10.1017/S0007114508026160 18613985

[34] Martinon F, Burns K, Tschopp J. The inflammasome: a molecular platform triggering activation of inflammatory caspases and processing of proIL-beta. Mol Cell. 2002; 10: 417–426. 12191486 10.1016/s1097-2765(02)00599-3

[35] Gessner DK, Gröne B, Rosenbaum S, Most E, Hillen S, Becker S, et al Effect of dietary fish oil on the expression of genes involved in lipid metabolism in liver and skeletal muscle of lactating sows. J Anim Physiol Anim Nutr (Berl). 10.1111/jpn.12324 [Epub ahead of print] 25865806

[36] Gessner DK, Gröne B, Rosenbaum S, Most E, Hillen S, Becker S, et al Treatment of lactating sows with clofibrate as a synthetic agonist of PPARα does not influence milk fat content and gains of litters. BMC Vet Res. 2015; 11: 54 10.1186/s12917-015-0368-y 25888880 PMC4355968

[37] Gessner DK, Fiesel A, Most E, Dinges J, Wen G, Ringseis R, et al Supplementation of a grape seed and grape marc meal extract decreases activities of the oxidative stress-responsive transcription factors NF-κB and Nrf2 in the duodenal mucosa of pigs. Acta Vet Scand. 2013; 55: 18 10.1186/1751-0147-55-18 23453040 PMC3599961

[38] Vandesompele J, De Preter K, Pattyn F, Poppe B, Van Roy N, De Paepe A, et al Accurate normalization of real-time quantitative RT-PCR data by geometric averaging of multiple internal control genes. Genome Biol. 2002; 3. 10.1186/gb-2002-3-7-research0034 PMC126239 12184808

[39] Ringseis R, Mooren FC, Keller J, Couturier A, Wen G, Hirche F, et al Regular endurance exercise improves the diminished hepatic carnitine status in mice fed a high-fat diet. Mol Nutr Food Res. 2011; 55 Suppl 2: S193–202. 10.1002/mnfr.201100040 21770048

[40] Samali A, Fitzgerald U, Deegan S, Gupta S. Methods for monitoring endoplasmic reticulum stress and the unfolded protein response. Int J Cell Biol. 2010; 2010: 830307 10.1155/2010/830307 20169136 PMC2821749

[41] Eckersall PD, Saini PK, McComb C. The acute phase response of acid soluble glycoprotein, alpha(1)-acid glycoprotein, ceruloplasmin, haptoglobin and C-reactive protein, in the pig. Vet Immunol Immunopathol. 1996; 51: 377–385. 8792574 10.1016/0165-2427(95)05527-4

[42] Heegaard PM, Klausen J, Nielsen JP, Gonzalez-Ramon N, Pineiro M, Lampreave F, et al The porcine acute phase response to infection with Actinobacillus pleuropneumoniae. Haptoglobin, C-reactive protein, major acute phase protein and serum amyloid A protein are sensitive indicators of infection. Comp Biochem Physiol B Biochem Mol Biol. 1998; 119: 365–373. 9629669 10.1016/s0305-0491(97)00362-3

[43] Hultén C, Johansson E, Fossum C, Wallgren P. Interleukin 6, serum amyloid A and haptoglobin as markers of treatment efficacy in pigs experimentally infected with Actinobacillus pleuropneumoniae. Vet Microbiol. 2003; 95: 75–89. 12860078 10.1016/s0378-1135(03)00136-6

[44] Cray C, Zaias J, Altman NH. Acute phase response in animals: a review. Comp Med. 2009; 59: 517–526. 20034426 PMC2798837

[45] Salamano G, Mellia E, Candiani D, Ingravalle F, Bruno R, Ru G, et al Changes in haptoglobin, C-reactive protein and pig-MAP during a housing period following long distance transport in swine. Vet J. 2008; 177: 110–115. 17509918 10.1016/j.tvjl.2007.03.015

[46] Delerive P, Gervois P, Fruchart JC, Staels B. Induction of IκBα expression as a mechanism contributing to the anti-inflammatory activities of peroxisome proliferator-activated receptor-α activators. J Biol Chem. 2000; 275: 36703–36707. 10980195 10.1074/jbc.M004045200

[47] Gessner DK, Schlegel G, Keller J, Schwarz FJ, Ringseis R, Eder K. Expression of target genes of nuclear factor E2-related factor 2 in the liver of dairy cows in the transition period and at different stages of lactation. J Dairy Sci. 2013; 96: 1038–1043. 10.3168/jds.2012-5967 23245956

[48] Hsu JM, Wang PH, Liu BH, Ding ST. The effect of dietary docosahexaenoic acid on the expression of porcine lipid metabolism-related genes. J Animal Sci. 2004; 82: 683–689. 10.2527/2004.823683x 15032424

[49] Feillet-Coudray C, Aoun M, Fouret G, Bonafos B, Ramos J, Casas F, et al Effects of long-term administration of saturated and n-3 fatty acid-rich diets on lipid utilisation and oxidative stress in rat liver and muscle tissues. Br J Nutr. 2013; 110: 1789–1802. 10.1017/S0007114513001311 23656726

[50] Yan YQ, Jiang W, Spinetti T, Tardivel A, Castillo R, Bourguin C, et al Omega-3 fatty acids prevent inflammation and metabolic disorder through inhibition of NLRP3 inflammasome activation. Immunity. 2013; 38: 1154–1163. 10.1016/j.immuni.2013.05.015 23809162

